# Chimpanzees spontaneously take turns in a shared serial ordering task

**DOI:** 10.1038/s41598-017-14393-x

**Published:** 2017-11-01

**Authors:** Christopher Flynn Martin, Dora Biro, Tetsuro Matsuzawa

**Affiliations:** 1 Department of Life Sciences, Indianapolis Zoo, Indianapolis, USA; 2 Department of Zoology, University of Oxford, Oxford, UK; 3 Institute for Advanced Study, Kyoto University, Kyoto, Japan

## Abstract

Social coordination can provide optimal solutions to many kinds of group dilemmas, and non-human subjects have been shown to perform single actions successively or simultaneously with partners to maximize food rewards in a variety of experimental settings. Less attention has been given to showing how animals are able to produce multiple (rather than single) intermixed and co-regulated actions, even though many species’ signal transmissions and social interactions rely on extended bouts of coordinated turn-taking. Here we report on coordination behaviour in three pairs of chimpanzees (mother/offspring dyads) during an experimentally induced turn-taking scenario. Participants were given a “shared” version of a computer-based serial ordering task that they had previously mastered individually. We found that minimal trial-and-error learning was necessary for the participants to solve the new social version of the task, and that information flow was more pronounced from mothers toward offspring than the reverse, mirroring characteristics of social learning in wild chimpanzees. Our experiment introduces a novel paradigm for studying behavioural coordination in non-humans, able to yield insights into the evolution of turn-taking which underlies a range of social interactions, including communication and language.

## Introduction

If “two heads are better than one”, it is primarily because those heads are capable of effectively coordinating their behaviour to achieve a common goal. Coordination between humans is known to be the optimal solution to many kinds of social dilemmas^1,2^, and conversational exchanges are central to cooperative problem solving^3^. Conversations require participants to take turns by temporally modulating their behaviour in accordance with others’ speaking and listening cues^4^. Turn-taking behaviours are also suggested to be an important feature of early language development^5^ and the acquisition of a theory of mind^6,7^. In addition to humans, many social species are known to rely on turn-taking processes to facilitate their signal transmissions and other collective activities^8–15^. Given the ecological importance of coordination in animal communication, as well as in human cognitive development and routine social interaction, a key challenge for comparative psychology is to determine the proximate mechanisms that underlie turn-taking behaviours across different species.

Among species that display a suite of coordinated behaviours, chimpanzees, the closest extant relatives of humans, are known in the wild to hunt cooperatively^16–18^, form coalitions to guard potential mates^19^, jointly patrol their territories^20^, and coordinate risky road crossings with dominant males acting as crossing guards^21^. In laboratory settings, chimpanzees have been shown to coordinate their behaviours to solve a variety of social dilemmas that require pairs of participants to perform one action each simultaneously^22,23^ or successively^24–28^. To examine coordinated turn-taking, additional interaction scenarios are needed that aim to require participants to make an extended series of inter-dependent actions in response to the behaviour of their partner.

We therefore developed a multiple-action task to test chimpanzees’ ability to coordinate actions in an experimentally induced turn-taking scenario. Three chimpanzee mother/offspring dyads were given a novel “shared” serial ordering task that required them to alternately press numerical stimuli in ascending order on a touch-panel screen. All participants were experts at such numerical ordering tasks from their prior experimental history^29,30^, involving stimulus sets comprised of consecutive as well as successive but non-consecutive numerals, but they had always completed them individually and had never experienced the shared version of the task.

Our results showed that the chimpanzees succeeded already in the earliest phases of the shared task with minimal trial-and-error corrections, suggesting that a propensity for coordinated turn-taking was already part of their social cognitive repertoire rather than the result of instrumental conditioning. Furthermore, offspring were quicker to take cues marking their turn from their mothers than vice versa, suggestive of asymmetries in attending to a partner’s behaviour as a function of dyadic role.

## Results and Discussion

**Figure 1:**
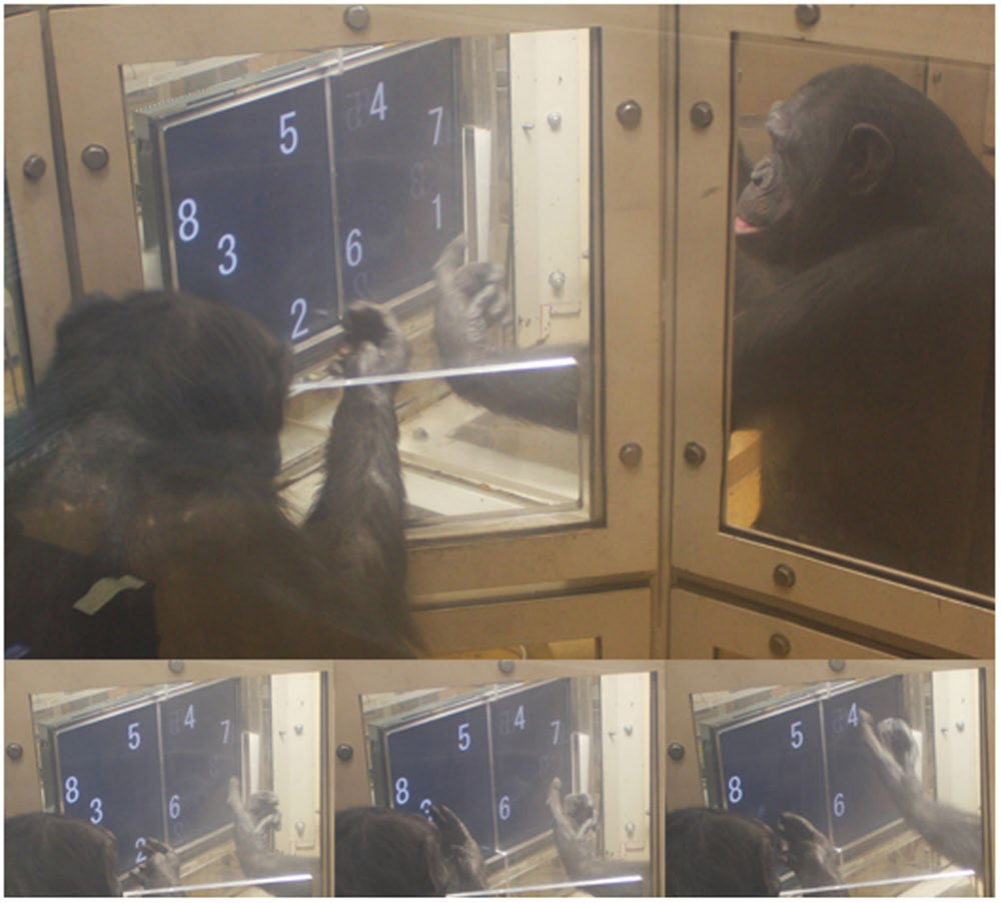
Still images illustrating progressive stages of a single trial of the serial ordering task. Two chimpanzees sit in adjacent booths and participate in a joint turn-taking task on a single touch-panel with a transparent barrier separating the two halves of the screen. The top panel shows the beginning of the trial, where the chimpanzee on the right is about to touch the first numeral in the sequence (1). In the bottom three panels, the numerals 2, 3 and 4 are being selected by the chimpanzee on the left, left and right, respectively. For temporal analysis, touch types were divided into “stays” (bottom middle) where the individual selecting a numeral does so after having selected the preceding numeral as well, and “switches” (bottom right), where the preceding numeral had been selected by the partner.

**Figure 2:**
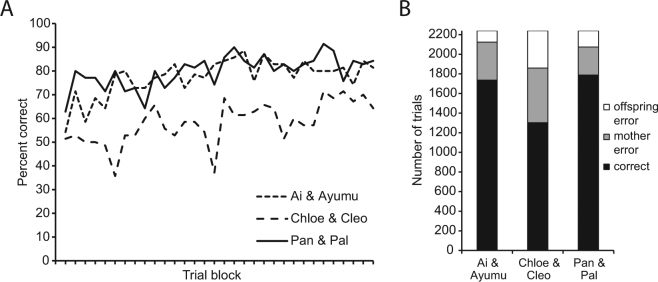
Accuracy in the eight-numeral ordering task. (**A**) Accuracy rates (percent of trials correct per session) of the three chimpanzee pairs over the 32 sessions of the eight-numeral task. (**B**) Total number of correct and incorrect responses for the 2240 trials given to each pair.

A single multi-touch monitor spanned across a common wall between two adjacent testing booths, giving the individual in each of the booths tactile access to half of the screen and visual access to the whole screen^31^. Numeral sets were presented at random locations across the whole of the screen, thereby requiring the subjects to alternately touch them in ascending order (Fig. 1; see also Supplementary Video S1). Initial exposure to the task was composed of an experimental phase with two numerals presented on the screen, followed by phases with three, four, and finally eight numerals (see Supplementary Methods for more detail). The pairs of subjects advanced through the initial three phases as their performance exceeded a predefined criterion level (higher than 80% correct responses on a rolling window of 24 consecutive trials). The criterion level of 80% was well above chance level, and was chosen to maintain consistency with the criterion levels used in prior numerical sequencing studies with these same subjects^32^. Three of the six subjects performed above the 80% criterion level from the first exposure to the task and maintained performance at that level or above throughout the initial three phases of the experiment (Table 1; Supplementary Table S1), suggesting spontaneous comprehension of the turn-taking heuristic. The final phase, involving eight-numeral stimuli sets (shown in Fig. 1), was given to subjects to assess their ability to adapt to a considerably more demanding turn-taking scenario. All three pairs performed with high accuracy from the outset of the eight-numeral task and further improved their performance over 32 repeated sessions (Fig. 2A). It should be noted that the chance level probability for successfully touching all eight numerals in ascending order on any given trial is one in 40,320.

**Table 1:** Mean number of trials needed to reach criterion accuracy (80%) beyond the first sliding window of 24 trials.

	Phase 1: Two Numerals				Phase 2:Three Numerals			Phase 3: Four Numbers	
Stage	1	2	3	4	1	2	3	1	2
Number Set	1-2	3-4	5-6	7-8	1-2-3	4-5-6	7-8-9	1-2-3-4	5-6-7-8
Ai	**47.5**	**12**	0	0	**20.3**	**3**	**0.5**	**2.9**	**1.6**
Ayumu	0	0	0	0	0	0	0	0	0
Chloe	**38**	0	0	0	**5.5**	**3.3**	**1.2**	**0.7**	**5.9**
Cleo	**9**	0	0	**1**	**6**	0	0	**2.8**	**6.5**
Pan	0	0	0	0	0	0	0	0	0.3
Pal	0	0	0	0	**0.5**	**1**	0	0	0

Note: Values represent the mean number of trials above the minimum number needed to reach criterion for all trial types in a given stage and phase. By definition, the minimum possible number of trials to reach criterion was 24. Zeros refer to cases when subjects achieved criterion-level performance in the first 24 trials of every trial type for a given phase and stage. Positive numbers in bold represent instances where subjects needed more than 24 trials to achieve criterion.

**Figure 3:**
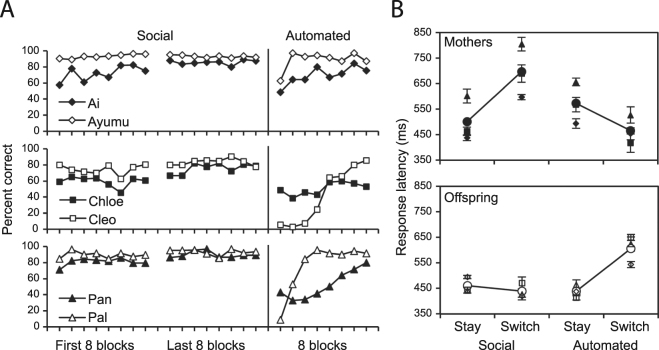
Accuracy and response latency comparisons. (**A**) Accuracy. Percent correct trials in the first and last eight blocks (out of the total of 32 blocks) of the 8-numeral social task are shown on the left. Percent correct trials during the first eight blocks of the subsequently introduced automated condition are shown on the right. Accuracy is calculated for each subject out of the total number of trials a subject received that were not terminated due to mistakes of their partner. (**B**) Response latency. Latencies reflect the length of time it took subjects to touch a target numeral in either of the following scenarios: (1) a “stay”, i.e., a touch made to a numeral by an individual after the same individual had touched the preceding numeral in the sequence, and (2) a “switch”, i.e., a touch made to a numeral by an individual after its partner had touched the preceding numeral in the sequence. Data (means ± s.e.m.) shown on the left are from the first eight and the final eight blocks of the 8-numeral social task (i.e., Phase 4 in the Supplementary Methods), and data shown on the right are from the eight blocks of the automated condition.

The use of mother/offspring dyads in this study allowed us to examine differences in behavioural strategies employed as a function of dyadic role. We compared overall performance rates (Fig. 2) and response latencies (Fig. 3B) between mothers and offspring. In all three pairs, offspring made fewer errors compared to their mothers (Fig. 2B; Supplementary Table S2) while both groups made similar kinds of errors (Supplementary Table S3). Response latencies were measured for two different types of touches to the screen: “stay touches”, referring to a touch made to a numeral by an individual after the same individual had touched the preceding numeral in the sequence, and “switch touches”, referring to a touch made to a numeral by an individual after the partner had touched the preceding numeral in the sequence (Fig. 1). The mean response latencies of stay and switch touches to seven numerical stimuli (2 through 8) for each subject were analysed by a linear mixed-effects model (LMM) with dyadic role (mother, offspring), touch type (stay, switch), numeral (2 through 8), and pair (1 through 3) as fixed effects, and subject ID as a random effect nested within dyadic role. The LMM revealed a significant main effect of numeral on response latency (t = −5.69, P < 0.001) and a significant interaction between dyadic role and touch type (t = 10.21, P < 0.001; see Supplementary Table S4 for detailed outputs of all LMM models). The significance of the interaction term was further confirmed through a maximum-likelihood comparison to a model without the interaction (χ2 = 66.22, P < 0.001). This interaction indicates that the response latency to switches was delayed for mothers, but not for offspring (“Social” condition in Fig. 3B). Besides confirming that the speed of touching increases as the number of stimuli remaining on the screen decreases, these results also suggest, crucially, that offspring were more efficient at responding to their mothers’ behaviour than the mothers were to the behaviour of their offspring.

Was this truly a “social” task? Without any real social coordination, animals can be trained to behave as if they modify their behaviour based on another individual’s response through simple conditional associations^33^. A central question in the interpretation of these results is therefore whether or not social cognitive processes were in fact responsible for the subjects’ seemingly coordinated behaviours. For the shared serial ordering task, a non-social strategy would have involved attending only to the disappearance of the stimuli from the other side of the screen, and not to the behaviour of the partner. To control for this possibility, the chimpanzees were given an automated version of the ordering task that was similar to so-called “ghost condition” methods traditionally used for dissociating social from non-social learning effects^34,35^. Subjects were tested without a partner in the adjacent booth while the numbers on the other side of the touch-screen disappeared automatically as dictated by a computer program (see Supplementary Methods for more detail). It was hypothesized that had the subjects been ignoring their partner’s behaviour and relying instead on the mere disappearance of the numerals during the social task, their accuracy rates, types of errors, and response latencies would not change significantly when confronted with the automated partner.

Contrary to the predictions of the non-social hypothesis, all subjects experienced a decrement in performance during the initial presentation of the automated condition (Fig. 3A). A repeated measures ANOVA showed that this drop was significant across the six subjects (F_1,90_ = 19.64, P = 0.007). Examining the data in Fig. 3A suggests that in the case of Ayumu and Pal the drop was more quickly recovered than among the other four subjects. Additional error analysis revealed that the types of errors made by subjects were different between conditions, which also suggested that observance of the partner’s actions had an effect on the subjects’ performance. The total number of errors made by each subject was divided into two error types: “stay errors”, which refer to mistakes made by touching an incorrect numeral while the correct target numeral was located on the subject’s side of the touch-panel, and “switch errors”, in which the target numeral was located on the partner’s side of the touch-panel when the subject touched incorrectly. Switch errors became more frequent during the automated condition for five of the subjects (Supplementary Figure S1; Chi-Square: Ai, χ² = 39.43, P < 0.001; Ayumu, χ² = 1.08, P = 0.29; Chloe, χ² = 4.23, P = 0.039; Cleo, χ² = 4.67, P = 0.03; Pan, χ² = 60.75, P < 0.001; Pal, χ² = 8.35, P = 0.003).

To determine if the timing of touching behaviour was affected by the partner’s direct involvement in the task, the mean response latencies of touch types (stay and switch) by each subject in both conditions (social and automated) were analysed statistically. A LMM on offspring response latency revealed a significant interaction between touch type and condition (t = 9.58, P < 0.001), and a second LMM on mothers also revealed an interaction between touch type and condition (t = −9.74, P < 0.001) but in a different direction, as evidenced by the opposite signs for the coefficients provided by the two models for the interaction term (Fig. 3B; see also Supplementary Table S4). In both cases, the significance of the interaction term was further confirmed through a maximum-likelihood comparison to a model without the interaction (χ2 = 62.00, P < 0.001 for offspring; χ2 = 62.78, P < 0.001 for mothers), and the difference between mothers and offspring in the direction of the effect through a model with the 3-way interaction term touch type * condition * dyadic role (t = −13.28, P < 0.001).

The differences in response latencies and performance rates between the social and automated conditions suggest that for both mothers and offspring the presence of a partner had significant, yet different, effects on their strategy for solving the task. An alternative explanation is that the differences may have largely been due to an unexpected change in procedure (removal of the partner), and that such a change was likely to have been enhanced by the higher amount of training and exposure to the social task compared to the automated task. This explanation cannot be ruled out, although steps were taken to keep the task itself as consistent as possible between the social and automated conditions (for example, the computer was programmed to precisely mimic the timing patterns of each specific partner), so that the major procedural change was limited to the absence of the partner’s behavioural cues. Along these same lines, a further consideration involving the partner’s absence was the possibility of confounding social facilitation effects, which would have posed an interference if the presence of the partner in the adjacent touch-panel booth had enhanced the ability of subjects to solve the task by increasing their arousal, motivation, attention, and engagement levels^36^. Efforts were made to mitigate the impact of such effects by keeping the partner during the automated condition situated in the immediate vicinity of the subject (within 3 meters and separated by transparent acrylic panel walls), albeit not in the adjacent touch-panel booth due to logistical constraints (e.g. had the partner been in the touch-panel booth, they would likely have disrupted the automated procedure by trying to touch the screen). Future studies that elaborate on the computerized partner method developed in this study may help to elucidate the impact of these potential confounds.

Shared instrumental actions over multiple bouts of behaviour have been previously examined in studies on other non-human primate species^37–39^, as well as in a recent study using chimpanzee pairs by Fletcher and colleagues^40^ that focused on the topic of role-comprehension. In that study, pairs of individuals alternated actions that caused a marble to roll between them through a shared puzzle box. The distributions of actions were always strictly alternating (self-other-self-other), and the location of the marble served as a salient cue for when each individual should act, so an examination of social turn-taking was limited. In the current study, there were seventy different permutations of self-other action sequences, and there was no simple external cue to guide behaviour, because the decision to press a given numeral at a given time needed to come from an associative link between the visual input of the numerals remaining on the screen, and the subjects’ internal representations of the numerals’ order.

Turn-taking in chimpanzees has also been examined within the context of reciprocal food sharing, with tasks involving a requisite alternation of token exchanges^41^ or specific rope pulls^42^ for achieving optimal food rewards. Chimpanzees in these studies failed to jointly establish a robust turn-taking heuristic across multiple trials, though it should be noted that rewards were delivered after each individual action. In the current experiment multiple actions were required of each individual before any food was dispensed.

For humans, informal verbal interactions constitute a core aspect of social life, and it follows that the empirical study of “turn-taking” has its origins in the fields of sociology and linguistics. Prior research in these fields has modelled the structure of conversational exchanges, compared them across cultures, and examined their neural and cognitive underpinnings^4,43,44^. Findings from these studies point to a set of turn-taking characteristics that are generally consistent across cultures^4^, such as the temporal distributions of verbal turns and the gaps between them^43^, as well as neural evidence of complex multitasking^44^, since during conversation the production of a response temporally overlaps with the comprehension of a turn. Such findings are suggestive of an innate interactional framework that underpins language use and development, and suggest that communicative turn-taking behaviours may be an evolutionary precursor to language^44^. Furthermore, recent work has begun to emphasize the importance of considering multimodal signal production and reception for a fuller understanding of turn-taking during social interaction in animal communication^45^.

Phylogenetic comparisons offer to further elucidate the evolutionary roots of turn taking in a communicative context. A recent study by Fröhlich and colleagues^46^ presented evidence for turn-taking in wild chimpanzee and bonobo mother/offspring dyads prior to joint travel episodes. The chimpanzees and bonobos observed used bidirectional gestural signals in immediate, overlapping, and delayed sequences during exchanges, and their cooperative turn-taking exhibited many similarities to human conversational exchanges. Extended bouts of alternating actions have also been observed during routine social activities of chimpanzees including reciprocal grooming, play chasing and wrestling^47,48^. Many other animal species whose communications are transmitted in a back-and-forth manner also display turn-taking coordination^8–14^. Within the primate lineage, it has been suggested by Takahashi and colleagues^14^ that the capacity for vocal turn-taking found in humans and marmosets may have resulted from a highly pro-social temperament composed of cooperative breeding habits, strong social bonding, high intragroup tolerance, cooperative problem-solving, and active food sharing. Chimpanzees display many of these prosocial tendencies^49^ (although the role of potential experimental artefacts in promoting these tendencies is currently heavily debated^50^), with the major exception being cooperative breeding, though some cases of alloparenting have been observed in the wild^51,52^. In light of a “by-product of selection for temperament” hypothesis^14^, the presence in chimpanzees of turn-taking for shared instrumental actions is consistent with a temperament that does not involve cooperative breeding habits but which is otherwise highly prosocial.

Our findings that offspring were particularly responsive to the behaviour of their mothers (more so than the reverse) echo previous observations on the existence of attentional asymmetries both in captivity^53^ and the wild^54^. Indeed, they are also in line with patterns of communicative turn-taking in wild chimpanzees and bonobos, where overlapping responses were found to be more common in infants and delayed responses were more frequent in mothers^46^. Furthermore, the findings may provide insights into both the proximate mechanism underlying the turn-taking behaviour itself, and its implications for group-level characteristics of social information flow in wild chimpanzee communities. First, in regards to the former, while the quick response latency of offspring during the social task may suggest that young chimpanzees were comparatively more adept at solving cognitive puzzles, the latency results from the automated condition (in which mothers were quicker than offspring on switch touches) oppose this notion. Instead, we suggest that this particular discrepancy might be related to the mothers’ longer experimental history with non-shared numerical sequencing tasks, which might have interfered with their ability to adapt to the shared version. A recent study^55^ on the neural mechanisms underlying human turn-taking behaviour during piano duets showed that transcranial magnetic stimulation to the dorsal premotor cortex disrupted players’ performance only if they had rehearsed their partner’s role beforehand, but not if no prior rehearsal had taken place. The authors suggest that coordinated turn-taking might therefore involve acting on predictions of what a partner will do based on one’s own experience with carrying out the action sequence in question (through a process referred to as “motor simulation”, i.e., anticipation of those actions in a sequence that are not carried out by oneself). In the current study, mothers with more experience than their offspring at completing numerical sequencing tasks may have been assigning a similar prediction heuristic to their offspring’s behaviour, and experienced greater temporal disruptions when their partners’ behaviour did not match the timing of their own extensively rehearsed actions.

Second, it is possible that the asymmetry in tracking social cues we documented is also an inherent property of age-related differences in chimpanzee social attention and cognition, and that its implications thus include complex community-level processes. For example, prior studies of wild chimpanzee material cultures have posited that directed social learning underlies the maintenance of community-specific tool use traditions^54,56–58^. Specifically, the “master-apprenticeship” hypothesis states that young chimpanzees acquire tool use behaviours through continual observation of their mothers, who perform those behaviours in sight of their offspring without actively teaching^57^. Results from the current study may shed light on how information flows in a similar manner through mother-offspring dyads that are not engaged in social learning per se, but are rather engaging in a joint endeavour that requires coordination over a sequence of actions. Possible future studies involving a greater variety of subject pairings (composed, for example of differential rank, kinship, or familiarity) should provide more conclusive insights into the precision, speed, and direction of social information flow within chimpanzee groups.

## Methods

### Subjects and experimental protocols

Six chimpanzees (*Pan troglodytes*) served as subjects: three mothers named Ai (age 35), Chloe (age 32), Pan (age 29), and their respective offspring, named Ayumu (male, age 12), Cleo (female, age 12), and Pal (female, age 12). None of the mothers had any other offspring, and none of the offspring were themselves mothers. Mothers were always paired with their own offspring throughout all experimental sessions. We used all available mother/infant pairs at the facility that were able to perform the task. We limited pairing to mother-offspring dyads for logistical reasons, as it best ensured that subjects would be willing to perform a joint task. While our sample size was small, it nonetheless provided three independent replicates for a highly sophisticated task that required substantial pre-existing competence at handling numerical sequences.

Subjects were members of a community of 13 chimpanzees at the Primate Research Institute of Kyoto University, where they lived in a semi-natural enriched enclosure. For experimental sessions, mother/offspring dyads voluntarily entered a twin testing booth, which had sliding doors connecting the two chambers, and acrylic panel walls on all sides (including between the two chambers). A single 22-inch touch-panel (3M C2254PW) was located along the joint wall of the twin booth such that each subject had tactile access to half of the screen, and visual access to the whole screen. Food rewards for correct trials (8 mm apple cubes) were delivered automatically by two universal feeders (Biomedica, BUF-301 P100). The experimental task required the participants to touch between two and eight numerals in ascending order. During the eight-numeral task, each subject was given four numerals to press, and every possible way of dividing the eight numerals into two groups of four and spreading them across the two halves of the screen was accounted for, resulting in seventy possible permutations or “trial types”. Some of these trial types followed a strictly alternating “back and forth” pattern, while others required subjects to touch multiple numerals in sequence before it was the other subject’s turn. The locations of numerals on each half of the screen were distributed randomly inside a 5-by-10 grid. For the eight-numeral task, each subject pair participated in 32 sessions of 72 trials each (social condition), followed by eight sessions with only one member of the pair (automated condition). In the automated condition, each subject participated individually, and numerals disappeared from the partner’s side automatically, according to a pre-programmed schedule. This schedule was based on the partner’s average response latency during the social condition for each number in each of the seventy possible trial types. All methods involving the use of chimpanzee participants for this study were carried out in accordance with the 3rd edition of the *Guide for the Care and Use of Laboratory Primates* issued by Kyoto University Primate Research Institute in 2010, and the experimental protocol was approved by the Animal Welfare and Animal Care Committee of the same institute.

### Statistical analysis

The effects of dyadic role (mother or offspring), response type (switch or stay) and experimental condition (social or automated) on subjects’ response latencies were analysed using Linear Mixed-effects Models (LMMs). Dyadic role, response type, numeral, pair, and condition were included as fixed effects, and subject ID was included as a random effect, nested in dyadic role. When models included interaction terms (dyadic role * response type, or response type * experimental condition), the significance of these terms were further assessed through maximum-likelihood comparisons to models containing the main effects but not the interactions. A repeated measures ANOVA was used to test for differences in subjects’ accuracy between the last eight blocks of the social condition and the first eight blocks of the automated condition. Response latency data were log-transformed and accuracy data were arcsine transformed to meet assumptions of parametric analysis; homogeneity of variance and normality of error were confirmed for all models following the transformations. All statistical analyses were performed in R (version 3.3.2; R Foundation for Statistical Computing, Vienna, Austria), using the *lme4* package^59^. The outputs of all models are included in the Supplementary Information (Supplementary Table S4).

## Electronic supplementary material

Supplementary Information

VideoS1
