# Supplementary material for: Chimpanzees spontaneously take turns in a shared serial ordering task
Source: Sci Rep. 2017 Nov 1;7:14307. doi: 10.1038/s41598-017-14393-x (PMC5665892; doi:10.1038/s41598-017-14393-x)
Supplement: Supplementary file 1 — Supplementary Information [file 41598_2017_14393_MOESM1_ESM.pdf]

Supplementary Information

Chimpanzees spontaneously take turns in a shared serial ordering task

Christopher Flynn Martin[1], Dora Biro[2], Tetsuro Matsuzawa[3]

[1]*Department of Life Sciences, Indianapolis Zoo, USA*

[2]*Department of Zoology, University of Oxford, UK*

[3]*Department of Brain and Behavioral Sciences, Kyoto University Primate Research Institute, Japan*

CORRESPONDENCE:

Christopher Martin

Indianapolis Zoo, 1200 West Washington Street, Indianapolis, Indiana 46222

Email: chris.f.martin@gmail.com

(317)630-2016 (Voice)

(317)630-5114 (Fax)

Supplementary Tables

Table S1. Number of trials required for each subject to reach criterion accuracy (80%) at each stage of experimental phases 1-3 of the serial ordering task.

Phase	Numeral Set	Trial Types	Ai			Ayumu			Chloe			Cleo			Pan			Pal		
			N	O	P	N	O	P	N	O	P	N	O	P	N	O	P	N	O	P
1	1,2	AA	26	5	-	24	0	-	100	54	-	24	0	-	24	0	-	24	1	-
		BA	117	-	67	24	-	2	24	-	4	42	-	12	24	-	2	24	-	3
	3,4	AA	24	3	-	24	0	-	24	0	-	24	0	-	24	0	-	24	1	-
		BA	48	-	21	24	-	0	24	-	2	24	-	0	24	-	0	24	-	4
	5,6	AA	24	0	-	24	0	-	24	1	-	24	4	-	24	1	-	24	2	-
		BA	24	-	3	24	-	2	24	-	2	24	-	0	24	-	0	24	-	3
	7,8	AA	24	2	-	24	1	-	24	0	-	26	6	-	24	0	-	24	3	-
		BA	24	-	2	24	-	4	24	-	3	24	-	4	24	-	3	24		2
2	1, 2,3	AAA	24	2	-	24	0	-	26	2	-	24	3	-	24	0	-	24	3	-
		AAB	24	0	-	24	0	-	24	0	-	24	1	-	24	0	-	24	0	-
		ABA	28	-	7	24	-	0	50	-	14	60	-	22	24	-	1	27	-	5
		BBA	39	-	8	24	-	0	24	-	4	24	-	0	24	-	0	24	-	0
		BAA*	103	**1**	**45**	24	1	0	26	2	4	24	0	0	24	0	2	24	0	0
		BAB	48	-	11	24	-	1	27	-	7	24	-	3	24	-	2	24	-	0
	4,5,6	AAA	24	3	-	24	1	-	24	4	-	24	1	-	24	0	-	24	0	-
		AAB	24	0	-	24	0	-	24	2	-	24	0	-	24	0	-	24	0	-
		ABA	24	-	4	24	-	0	24	-	1	24	-	3	24	-	0	24	-	2
		BBA	24	-	0	24	-	2	24	-	2	24	-	1	24	-	0	26	-	0
		BAA*	42	**0**	**16**	24	1	1	26	0	5	24	1	1	24	1	0	29	1	5
		BAB	24	-	2	24	-	0	42	-	15	24	-	0	24	-	0	26	-	5
	7,8,9	AAA	24	0	-	24	0	-	24	0	-	24	0	-	24	1	-	24	1	-
		AAB	24	0	-	24	0	-	24	0	-	24	1	-	24	0	-	24	0	-
		ABA	24	-	3	24	-	0	24	-	0	24	-	1	24	-	0	24	-	0
		BBA	24	-	0	24	-	1	24	-	1	24	-	2	24	-	2	24	-	2
		BAA*	27	**0**	**6**	24	0	0	25	0	5	24	0	1	24	1	0	24	0	0
		BAB	24	-	2	24	-	0	30	-	7	24	-	1	24	-	0	24	-	0
3	1,2,3,4	AAAA	24	0	-	24	2	-	24	3	-	24	1	-	24	1	-	24	1	-
		BAAA*	45	2	9	24	0	0	24	0	3	24	0	3	24	0	0	24	1	0
		AAAB	24	0	-	24		-	24	2	-	24	0	-	24	0	-	24	3	-
		BBBA	24	-	0	24	-	0	24	-	1	24	-	0	24	-	1	24	-	0
		AABB	24	0	-	24	0	-	24	0	-	24	0	-	24	0	-	24	1	-
		BBAA*	24	1	2	24	0	0	24	0	0	24	0	1	24	0	0	24	0	0
		ABAA*	24	1	2	24	0	1	24	0	3	24	0	4	24	1	0	24	0	1
		BABB	24	-	1	24	-	0	24	-	4	24	-	2	24	-	0	24	-	1
		AABA*	34	0	9	24	0	2	34	0	6	64	1	27	24	1	0	24	0	0
		BBAB	24	-	0	24	-	0	24	-	0	24	-	0	24	-	0	24	-	0
		BAAB*	25	0	5	24	0	0	24	0	4	24	0	2	24	0	1	24	0	3
		ABBA	24	-	0	24	-	0	24	-	0	24	-	1	24	-	0	24	-	2
		ABAB	24	-	0	24	-	1	24	-	3	24	-	0	24	-	1	24	-	0
		BABA	33	-	5	24	-	0	24	-	3	24	-	4	24	-	3	24	-	0
	5.6.7.8	AAAA	24	1		24	1	-	24	4	-	25	5	-	24	4	-	24	1	-
		BAAA*	26	2	3	24	0	0	24	0	3	24	4	1	26	1	4	24	2	0
		AAAB	24	0	-	24	0	-	24	1	-	24	2	-	24	0	-	24	1	-
		BBBA	24	-	2	24	-	2	24	-	0	24	-	1	24	-	1	24	-	0
		AABB	24	0	-	24	1	-	24	0	-	24	1	-	24	0	-	24	0	-
		BBAA*	24	1	2	24	0	0	24	0	1	24	0	0	24	1	0	24	0	0
		ABAA*	24	1	2	24	0	2	45	0	11	24	0	2	24	0	1	24	0	0
		BABB	24	-	4	24	-	0	24	-	4	24	-	1	24	-	1	24	-	0
		AABA*	24	0	3	24	0	0	31	2	3	86	1	20	24	1	2	24	0	0
		BBAB	24	-	2	24	-	0	24	-	1	24	-	0	24	-	0	24	-	0
		BAAB*	44	4	6	24	0	0	24	2	2	24	0	3	26	2	3	24	0	0
		ABBA	24	-	0	24	-	1	24	-	1	24	-	0	24	-	1	24	-	1
		ABAB	24	-	1	24	-	0	50	-	11	24	-	3	24	-	0	24	-	0
		BABA	24	-	3	24	-	1	52	-	13	52	-	11	24	-	0	24	-	0
		Total	1621	29	258	1296	8	23	1524	79	153	1483	32	137	1300	16	31	1308	22	39

Note: In the Trial Types column, the letter A represents touches made by the focal subject and B represents touches made by the focal subject's partner. For example, in the final row, for the trial type BABA containing 5-6-7-8 as stimuli, the order of touches to numbers was: partner (5), subject (6), partner (7), subject (8). Column N shows total number of trials required by each subject to reach the criterion level, and columns O and P show errors made while the target numeral was on the subject's own side of the monitor (column O), and errors made when the target numeral was on the partners side of the monitor (column P).

Table S2. Social task within-pair error analysis.

	Incorrect responses			
**Pair**	**Mother**	**Offspring**	**Chi-Square**	**P**
Ai /Ayumu	383	119	138.837	<0.001*
Chloe /Cleo	560	377	35.741	<0.001*
Pan /Pal	295	156	42.840	<0.001*

Note: The 'Incorrect responses' columns, separated into errors by mothers and offspring, refer to the number of trials (out of the total 2240 trials presented) in which the subject touched a numeral other than the one that came next in the sequence.

Table S3. Social task within-subject error analysis.

		Incorrect responses			
**Subject**	**Age class**	**Target on own side**	**Target on partner's side**	**Chi-Square**	**P**
Ai	Mother	98	285	91.303	<0.001*
Chloe	Mother	116	444	192.114	<0.001*
Pan	Mother	91	204	43.285	<0.001*
Ayumu	Offspring	52	67	1.891	0.169
Cleo	Offspring	122	255	46.920	<0.001*
Pal	Offspring	61	95	7.410	0.006*

Note: The 'Incorrect responses' columns refer to the number of trials (out of the total 2240 trials presented) in which the subject touched a numeral other than the one that came next in the sequence. **'**Target on own side' refers to incorrect touches made while the correct target numeral was on the focal subject's side of the touch-panel (i.e., the subject skipped a numeral on its own side of the screen), and 'Target on other side' refers to incorrect touches made while the correct target numeral was on the partner's side of the touch-panel (i.e., the subject failed to wait for the partner).

Table S4. Summary of Linear Mixed-effect Model (LMM) outputs. The table lists results for four LMMs with *dyadic role* (mothers, offspring), *touch type* (stay, switch), *numeral* (2 through 8), experimental *condition* (social, automated), and *pair* (1 through 3) as fixed effects, and *subject ID* as a random effect (nested within *dyadic role* in models including both mothers and offspring). The response variables were log-transformed response latencies, and in each model only the highest-order interaction term is considered (lower-order terms are listed in grey). Data are from the social condition (Model 1) and from both the social and automated conditions (Models 2-4).

Model number	Response variable	Predictors	Estimate	SE	t	P
1	Response latency	*Intercept*	6.204	0.155	40.008	<0.001
		dyadic role	-0.192	0.082	-2.334	0.020
		touch type	-0.358	0.051	-6.988	<0.001
		numeral	-0.023	0.004	-5.690	<0.001
		pair	0.096	0.041	2.346	0.019
		*dyadicrole*touchtype*	0.330	0.032	10.213	<0.001
2	Offspring response latency	*Intercept*	6.683	0.100	67.068	<0.001
		touch type	-0.420	0.010	-6.867	<0.001
		condition	-0.419	0.061	-6.854	<0.001
		numeral	-0.017	0.005	-3.548	<0.001
		*touchtype*condition*	0.370	0.039	9.577	<0.001
3	Mother response latency	*Intercept*	5.192	0.158	32.844	<0.001
		touch type	0.880	0.089	9.900	<0.001
		condition	0.684	0.089	7.688	<0.001
		numeral	-0.001	0.007	-0.161	0.872
		*touchtype*condition*	-0.548	0.056	-9.737	<0.001
4	Response latency	*Intercept*	7.911	0.287	27.559	<0.001
		touch type	-1.720	0.173	-9.959	<0.001
		condition	-1.522	0.173	-8.804	<0.001
		dyadic role	-1.411	0.178	-7.938	<0.001
		numeral	-0.009	0.004	-2.115	0.034
		pair	0.071	0.027	2.599	0.009
		*touchtype*condition*	1.288	0.109	11.784	<0.001
		*touchtype*dyadicrole*	1.300	0.109	11.893	<0.001
		*condition*dyadicrole*	1.103	0.109	10.087	<0.001
		*touchtype*condition*dyadicrole*	-0.918	0.069	-13.276	<0.001

Supplementary Figures

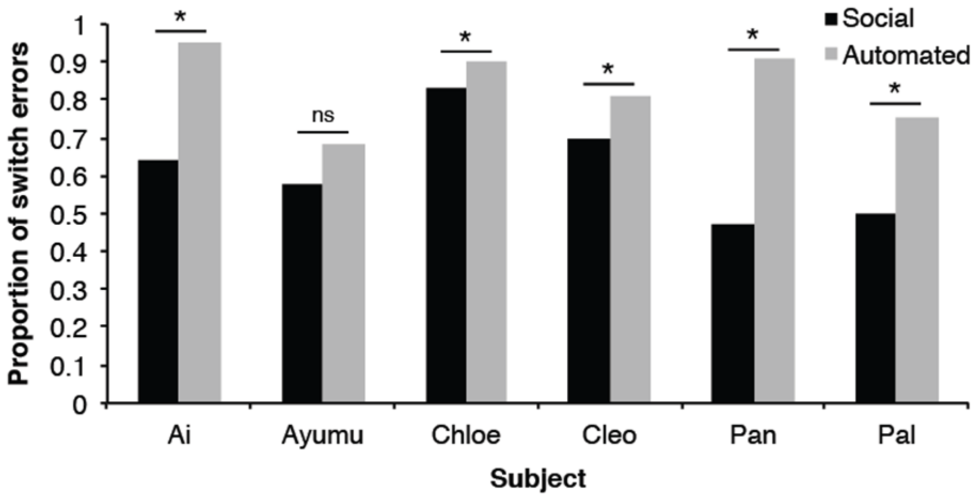

Figure S1. Comparison between social and automated error types.

Note: Errors on the task were separated into two categories: those which the target was located on the partner's side of the screen when the subject made a mistake (grey bars), and those in which the target was located on the subject's side of the screen when the subject touched incorrectly. Asterisks indicate significant differences ($P < .05$, Chi Square Test).

Supplementary Methods

Pairs of chimpanzees were called into the laboratory from their outdoor enclosure, and were led one by one into the two adjacent but separate experimental booths (see main text for further information on size of and touch-screen equipment installed in each booth). Once subjects were settled inside their respective booths, we activated the joint numerical sequencing task. Each trial commenced with the presentation of a small white circle in the outer lower corner of each half of the screen. Both subjects were required to touch this "start key", thus initiating the presentation of the numerical stimuli. Subjects were then shown numerals at random locations within a 4-by-8 matrix on the screen, and were required to respond by touching these numerals in ascending order. Upon being touched, a numeral disappeared from the screen. After the numerals were all touched in the correct order, a chime sounded and a food reward (small cube of apple) was delivered to both subjects simultaneously. If the numerals were touched in the incorrect order (i.e. a higher number touched before a lower one had been touched), a buzzer sounded, no food was delivered, and the trial was terminated.

Phase 1. In the first phase, only two numerals were presented to the subjects on each trial. Sessions consisted of 48 trials, and two sessions were carried out each day for each pair of participants. The 48 trials in a session were comprised of four trial types: the first two trial types

consisted of both numerals being presented on the same half of the screen, and the second two trial types consisted of the two numerals being divided across the separate halves of the screen. The four trial types were distributed in a pseudorandom order, such that no one trial type was presented more than three times in a row. The first phase was divided into four stages. In the first stage, the numerical stimuli consisted of numbers 1 and 2. The second stage consisted of numbers 3 and 4, the third stage consisted of numbers 5 and 6, and the fourth stage consisted of numbers 7 and 8. Progress from stage to stage depended on individual subjects' ability to reach an 80% correct criterion level. The 80% criterion was said to have been achieved when subjects performed at or above that level of accuracy within a sliding window of 24 consecutive trials of each trial type. After both subjects in a given pair passed the criterion level for each of the trial types, the pair moved on to the next stage. As the minimum number of trials that were presented before a subject could reach the criterion level in each trial type was 24, if a subject gave correct responses to 20 or more of the initial 24 trial presentations (i.e., was at or above 80% correct), its ability to complete the task was said to be spontaneous rather than the result of trial-and-error learning.

Phase 2. In the second phase, three numerals were presented. As in the first phase, sessions consisted of 48 trials, and two sessions on average were given each day to each pair of participants. The 48 trials in a session were divided into eight trial types: the first two trial types consisted of all three numerals being presented to a single subject on one half of the screen, and the last six trial types consisted of the numerals being divided across both halves of the screen, in all possible configurations. The eight trial types were distributed pseudo-randomly such that no one trial type was presented more than two times in a row. The second phase was divided into three stages. In the first stage, the numerical stimuli consisted of the number set 1-2-3. The second stage consisted of the number set 4-5-6, and the third stage consisted of the number set 7-8-9. As in the first phase, we set the criterion level at 80% correct over a sliding window of 24 trials for each of the eight trial types. Progress from one stage to the next was made as soon as both subjects of a pair reached criterion for all of the trial types.

Phase 3. In the third phase, four numerals were presented. The number of possible permutations of four numerals spread across two halves of the screen was 16 – due to this large number of possible trial types, we increased the number of trials per session to 96. Two sessions were given to each pair of participants on each day. The third phase was divided into two stages. In the first stage, the numerical stimuli consisted of the number set 1-2-3-4, and in the second stage of the number set 5-6-7-8. As in the first two phases, we set the criterion level at 80% correct over a sliding window of 24 trials for each trial type. Progress from the first to second stage was made as soon as both subjects of a pair reached criteria on all trial types.

Phase 4. After the subjects had completed the initial three phases, the fourth phase consisted of presenting eight numerals at random locations on the screen, with the constraint that the two halves always displayed four numerals each, resulting in a set of 70 possible permutations (trial types). 32 blocks were given with each block containing one each of the 70 possible trial types presented in random order. Data from trials in which subjects failed to complete all eight touches to numerals in the correct order were discarded from subsequent response latency analyses.

Automated Phase. Following Phase 4, subjects were given an 8 block automated condition, which was procedurally equivalent to the 8 numeral task in Phase 4 except for the absence of a conspecific partner (the partner was still present in the laboratory room, but was situated in a different booth from the shared touch-panel station). Instead of the partner's touches causing numerals to disappear on the opposite side of the panel from the subject, the numerals disappeared automatically. The computer was programmed to automatically 'touch' (i.e. erase from the screen) each numeral in turn on the partner's side according to timings equivalent to the mean response latency of the subject's real partner for each numeral in each trial type (70 trial types and four partner numerals in each trial type, for a total of 280 unique values programmed for each subject). In other words, trial progress was identical to the social condition, except for the absence of an actual live partner in the booth adjacent to the subject's and a simulation of the partner's touching behavior being provided by the computer. Subjects performed eight blocks of 70 trials each in this automated control condition.

Supplementary Video

Supplementary Video S1. A pair of participants, Ai and Ayumu, complete the shared serial ordering task with eight numerals presented on the touch-panel.
